# Integration of Metal Oxide Nanowires in Flexible Gas Sensing Devices

**DOI:** 10.3390/s130810659

**Published:** 2013-08-15

**Authors:** Elisabetta Comini

**Affiliations:** Sensor, Dipartimento di Ingegneria dell’informazione, Università di Brescia and CNR-IDASC, via Valotti 9, Brescia 25133, Italy; E-Mail: comini@sensor.ing.unibs.it; Tel.: +39-0-303-715-875; Fax: +39-0-302-091-271

**Keywords:** nanowires, gas sensors, flexible sensors

## Abstract

Metal oxide nanowires are very promising active materials for different applications, especially in the field of gas sensors. Advances in fabrication technologies now allow the preparation of nanowires on flexible substrates, expanding the potential market of the resulting sensors. The critical steps for the large-scale preparation of reliable sensing devices are the elimination of high temperatures processes and the stretchability of the entire final device, including the active material. Direct growth on flexible substrates and post-growth procedures have been successfully used for the preparation of gas sensors. The paper will summarize the procedures used for the preparation of flexible and wearable gas sensors prototypes with an overlook of the challenges and the future perspectives concerning this field.

## 1. Introduction

Moore's Law for the advancement of integrated circuits has been followed since the 1960s. Nowadays researchers in the microelectronics industry are trying to push nanolithography technology to its maximum in order to reduce the footprint of electronic devices, while battling its intrinsic limitations in the process. The emergence of nanowires as building blocks of multi-functional devices has already brought fundamental changes to the future of the IC industry and will possibly allow keeping up with Moore's Law. Nanowires can have very promising applications, primarily in logic circuits, but also as sensing and active elements for the development of highly sensitive bio/chemical/photon sensors. It is a critical step to afford reliable and economic scaled-up processes that may integrate nanowires into electronic devices. This challenge needs to be urgently met if nanotechnology is to evolve beyond simple academic interest. In particular the integration of nanowires into flexible substrates will further enable wearable devices for the monitoring of human health and welfare in real time twenty-four hours a day.

The controlled catalytic growth of semiconductor whiskers, and more recently nanowires, was discovered by Wagner and Ellis in 1964 [1], Si whiskers were grown by heating a Si substrate covered with Au particles in a mixture of SiCl_4_ and H_2_ and their diameter was determined by the size of Au particles. More than 20 years later Hitachi scientists applied this technique to the growth of III-V nanowhiskers [2]. Carbide [3] and oxide nanorods [4] were produced through vapor phase conversion and transport processes in several of these early studies.

**Figure 1.**
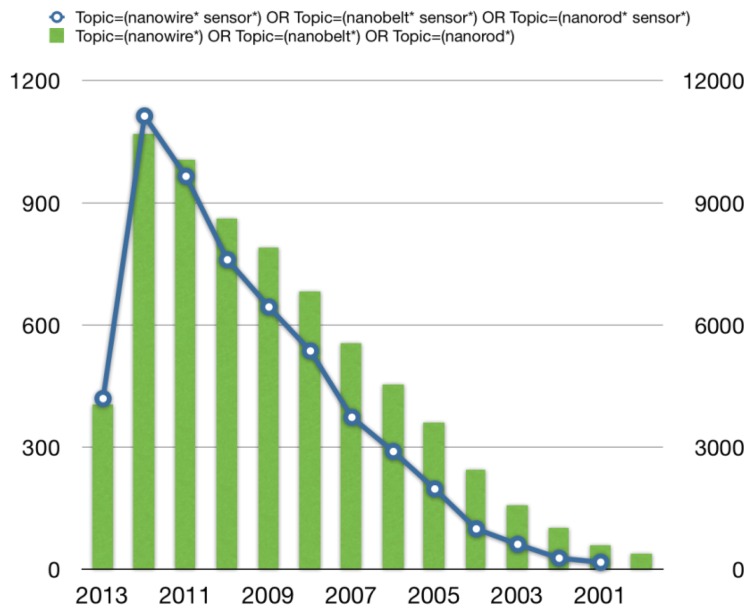
Number of publications as a function of the year from the isiwebofknowledge database 10/06/2013. Right axis: nanowires publications; left axis: nanowires sensors publications.

In 2000, the field of semiconductor nanowires underwent a significant expansion and became one of the most active research areas within the nanoscience community [5]. Stimulating improvements have been made at a surprisingly fast rate in different laboratories all over the World following curiosity-, discovery- or hypothesis-driven research. The field of nanowires remains an emerging research frontier. Researchers are constantly trying to acquire new fundamental knowledge on these nanomaterials while proposing novel potential applications. The trend in nanowires and nanowires sensors may be easily recognized looking at the increasing number of publications focused on nanowires and nanowire sensors in the last ten years, as reported in Figure 1.

Nanowires have proven applications in the field of electronics, photonics, energy conversion, energy storage, sensors, biosensors and also as interfacing tools between inorganic matter and living cells. Focusing on the sensing applications of nanowires, nanorods or nanobelts, the ten most cited articles (reported in Table 1) deal with the detection of chemical and biological species or nanoscale photonics and electronics. These were actually the first applications proposed and lead the worldwide research in the following years.

Several reviews have been published by different authors concerning the synthesis techniques and properties of these emerging nanostructures [6–12] but this research field is growing so fast that there are always new and significant advances and distinct approaches that were not covered in the previous reports.

This review is meant to focus on new research advances in flexible metal oxide semiconducting nanowires gas sensors expressing the author's personal interests and insights, while looking ahead to future directions.

Gas sensors may detect the presence of chemical or biological substances, they may be used for monitoring industrial processes, spoilage of food and toxic reagents along production lines, and moreover for ubiquitous control of the exposure to chemicals that may have negative effects on health. The measured gases in general are complex mixtures consisting of different molecules therefore arrays of sensors or electronic olfaction systems must be used.

Nanowires are interesting active materials for gas sensing for several reasons: very large surface to volume ratio, downsizing of the sensing materials, which improves the sensing performance, their stability thanks to their high degree of crystallinity, the simple and low cost preparation methods, the possibility of selective functionalization of the surface, and the ability to accommodate strain in case of lattice mismatch. The challenge is the integration of these nanostructures in macroscopic devices in a reliable way, with good and stable electrical contacts.

The advances made in recent years in fabrication technologies may allow the preparation of nanowires on flexible substrates. There are several advantages in using flexible transducers, for example light weight, foldability, transparency, wearability conformal coverage, and large-scale production. Flexible sensors have a great potential in a cost effective production for large-scale applications. Furthermore a decrease in the power consumption, interesting for the specific case of gas sensors, may be achieved using flexible microhotplate substrates. This broadens the range of potential applications. The main challenges are the elimination of high temperature steps in the device preparation process, and the stretchability of the all device including active materials and necessary circuitry. All these requirements must be addressed carefully for the achievement of reliable flexible gas sensors.

Among flexible sensors, wearable ones are the most interesting for a widespread monitoring of toxic compound and medical applications. Since the early development of wearable electronics, the biomedical application of sensors in diagnosis, therapy control or rehabilitation monitoring was clear. A wearable system typically consists of sensors, signal processing and transmission circuitry, power supply, and actuators. This field of research is attracting more and more interest nowadays, especially with regard to point-of-care diagnostics. The introduction of devices on flexible substrates has the advantage of mechanical flexibility during their operation, and furthermore the possibility to use a continuous roll-to-roll fabrication that can lead to an appreciable reduction in the production costs. This, in turn, will assure the widespread sensor use in all the aspects of real-time environmental monitoring, outdoor and/or indoor, for a better quality of life. In order to produce wearable devices with nanowires, they must be grown on flexible substrates or transferred after the growth. The more complex structures involved in gas sensors determines a large number of requirements: the active material has to be exposed to the environment for the sensing transduction, the device must withstand an abrasive weaving process and exposure to environmental contamination during its lifetime without modifying the sensing properties, it has to be mechanically robust and withstand repeated bending stresses, while remaining gas permeable, the power consumption must be reduced to avoid heating effects, and last but not least, it has to ensure and maintain wearing comfort.

## 2. Nanowires Integration

Integrating inorganic nanowires with plastic substrates, that are among the most attractive type of flexible substrate suitable for different applications, requires the use of process temperatures below the plastic glass transition or thermal degradation temperatures. Whereas organic materials are much more easily integrated into plastic substrates, their performance does not compare still to that of inorganic materials. Furthermore, long-term stability and reliability is a major concern. Besides the biggest challenge is to prepare highly crystalline materials at the temperatures that plastic can withstand. Apart from plastic, another interesting flexible substrate is paper, which is environmentally friendly, adaptable, cheap, biocompatible, lightweight, ubiquitous available, has a high surface to volume area due to the microfiber composition, a strong adhesion to a variety of materials and may be cut using standard low cost techniques.

For the device integration one can choose so far between two approaches: control the growth process itself inducing a selective deposition or make use of post-growth processes. The limitations for flexible/wearable devices preparation using the former approach are the maximum operation temperature and eventually the limitations in precursors and solvents that the substrate may withstand. The key factor for selective site deposition is the use of a catalyst-assisted deposition, pattering at specific locations onto the substrate, together with the use of specific gas flow, electric field or crystal surface selection.

### 2.1. Direct Growth on Flexible Substrates

Several experimental techniques to prepare metal oxide nanowires have been currently proposed and are well established, but just few allow the direct integration on flexible substrates. In general nanowires are grown on rigid substrates and then transferred onto flexible substrates, although there are some examples of nanowires grown directly on flexible substrates.

Vertically aligned ZnO nanowires [23] were grown by electrodeposition on transparent polyethylene terephtalate (PET) foil coated with indium tin oxide using a standard three-electrode electrochemical setup with a saturated Ag/AgCl reference electrode and a Pt foil as a counter electrode [24]. The electrodeposition was performed at 80 °C in an aqueous electrolyte containing ZnCl_2_, AlCl_3_ and KCl. For the generation of oxygen-saturated electrolyte solution, oxygen bubbling and magnetic stirring were used [25]. After one hour of deposition, a homogeneous coverage of vertically oriented ZnO nanowires is obtained. Using this simple deposition technique metal oxide nanowires were integrated into flexible light emitting devices. Other examples of fruitful integration on plastic substrates have been reported, in 2011 pure ZnO nanowires arrays and ZnO:Al nanostructures were grown on PET [26]. ZnO seed layers were prepared spinning an zinc acetate dihydrate-1-propanol solution on PET followed by an annealing at 100 °C, afterwards the substrates were dipped in a mixture of zinc nitrate hexahydrate and hexamethylenetetramine solution in deionized water. The hydrothermal process was conducted with microwave sintering system (2.45 GHz, 140 W) at atmospheric pressure. The use of a uniform seed oxide layer allowed the formation of well-aligned nanowires by rapid hydrothermal synthesis.

**Figure 2.**
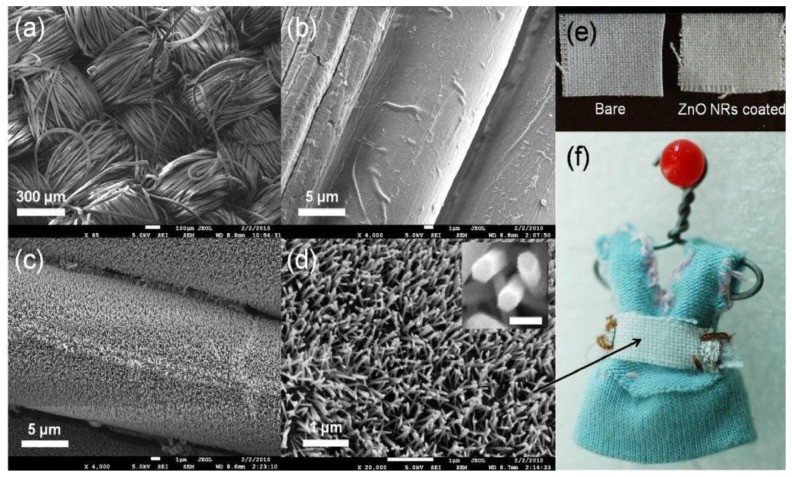
SEMimages of (**a**) cotton fabric substrate in low magnification; (**b**) bare cotton fibres; (**c**) ZnO NR-coated cotton fibres and (**d**) high magnification images of ZnO NRs on a cotton fibre. Scale bar in the inset represents 100 nm; (**e**) Photograph bare and ZnO NRs coated fabric; (**f**) Ready-to-wear ZnO NRs-on-fabric multifunctional sensing device sewn on a toy dress. Reprinted from [27] with permission. Copyright Elsevier (2010).

Moreover in 2010 the growth of ZnO nanorods has been performed directly on fabric in an easy, low cost, temperature and scalable process [27]. Even in this case a zinc oxide seed layer have been deposited, by sputtering, and the nanorods were prepared by hydrothermal technique with a growth solution containing an equimolar aqueous mixture of zinc nitrate, hexamethylenetetramine and poly(ethylenimine). The fabric was immersed in the growth solution and heated at 90 °C for 6 h and then washed in ethanol. Figure 2 reports the SEM images of the fabric and the nanowires together with the pictures of the fabric before and after the deposition process and the final device.

Recently a thermal oxidation technique for the direct integration of metal oxide nanowires on flexible and low power micro hotplates devices has been proposed [28–30]. Thermal oxidation consists in a two step process, first the deposition of a metal layer onto the desired substrates followed by a thermal oxidation treatment at atmospheric pressure in a oxygen-argon mixed environment at relatively low temperatures (200–450°). The mechanical stress produced by the temperature gradient and lattice mismatch between the metal and the oxide layer promote the formation of metal-oxide nanowires [31]. The advantages of thermal oxidation are the easy patterning procedure by shadow masking techniques, its reliability and reproducibility, the high production yield and its scalability for mass production. The drawbacks are the long time required for the growth (several hours) and the eventual presence of a residual layer beneath the nanowires.

**Figure 3.**
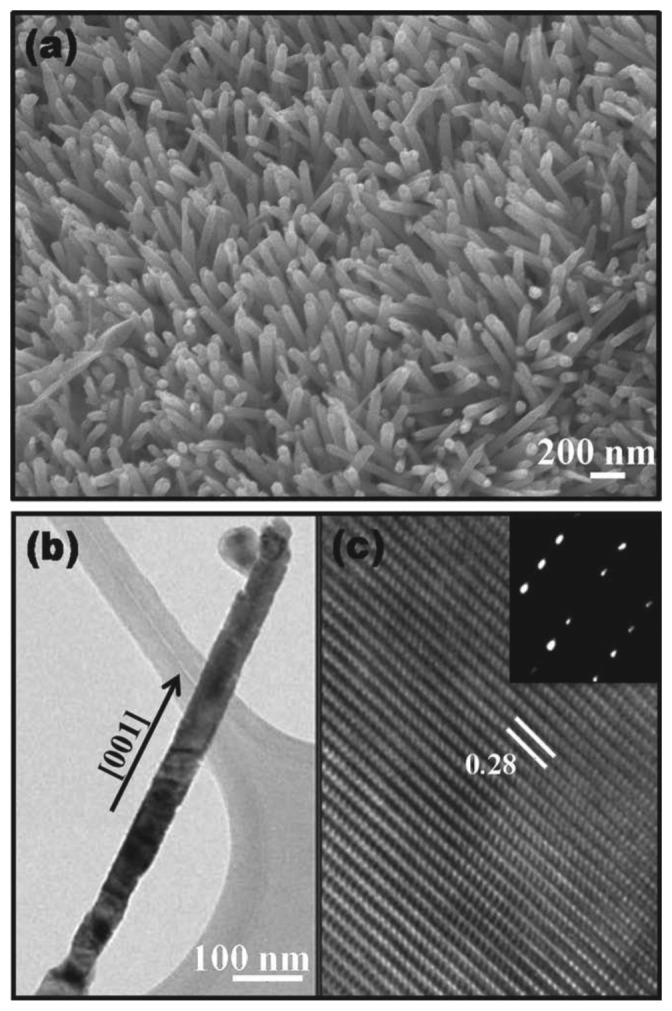
(**a**) Representative FESEM image of aligned ZnO nanorods; (**b**) TEM image of a single nanorod; (**c**) HRTEM image taken from the edge of the ZnO nanorod. Inset: Corresponding SAED pattern. Reprinted from [32] with permission. Copyright WILEY-VCH Verlag GmbH & Co. KGaA (2010).

The direct growth of nanowires on paper substrates is limited by the strict requirements of this flexible substrate, but recently some methods for the integration in functional devices on paper have been proposed in the literature. In the majority of these works the preparation of oxide nanowires/nanorods was performed again using a low temperature hydrothermal technique [32]. The results obtained are quite good in terms of crystallinity of the nanostructures as can be seen from Figure 3.

Most of the literature reporting a direct growth of metal oxide nanowires on flexible substrates is on zinc oxide, but there are several oxides interesting for chemical sensors, such as tin, indium, titanium and tungsten oxides that may be used for gas sensors development.

### 2.2. Post Growth Procedures

As far as the post-growth strategies are concerned, there are different approaches: wet transfer, dry-transfer or nanomanipulation. Nanowires, after the deposition procedure, are transferred to a receiver substrate and assembled by nanomanipulation, contact-printing, microfluidics, Langmuir-Blodget, flow assisted and electric field alignment. The most important thing to have in mind for a real world application is the possibility of a large scale production, and most of the abovementioned transfer techniques can satisfy this requirement on almost any substrate ranging from crystalline wafers to plastics.

Wet transfer consists in the dispersion of nanowires into a solvent to form a solution that is afterwards drop-cast onto the flexible substrate at ambient temperature. The disadvantages are lack of alignment and control in the nanowires arrangement, which can be overcome using electric field alignment as proposed in [33]. Dielectrophoretic assembly has been increasingly proposed for nanowires, thanks to the precise positioning ability possible on different substrates. In order to allow potential large-scale production, the yield must be very high; lately a 98.5% single nanowire yield on 16,000 electrodes over 400 mm^2^ has been demonstrated, which is quite impressive. The key forces that one has to balance for the achievement of such successful results are dielectrophoretic, hydrodynamic and surface forces. The nanowire-containing solution was put in contact with the substrate by a thin channel, and the stability of nanowires on an electrode was controlled by the complex nanowire–nanowire interactions, the interaction of the nanowires with the electrodes, and the hydrodynamic drag. Nanowire assembly can be made self-limiting, thus, single nanowires can be assembled on each electrode by carefully controlling the hydrodynamic and dielectrophoretic forces, the limits of this process is the ability of maintaining their uniformity. This method is promising for a large-scale nanomanufacturing process.

In 2007 a dry transfer method was proposed for silicon nanowires grown by the superlattice nanowire pattern transfer approach. This methodology works thanks to the deposition of nanowires on silicon-on-oxide wafers. The silicon oxide exposed is etched and a piece of poly(dimethylsiloxane) (PDMS) is put into contact with the nanowires, peeled from the substrate and attached to the transfer plastic substrate covered with a epoxy, finally the epoxy is cured and PDMS layer is peeled back leaving the nanowires on the transfer substrate. In the specific case of oxides nanowires this methods may present some problems related to the silicon dioxide etching process that can have effects also on metal oxide nanowires.

Later a contact printing technology has been proposed for nanowires and it seems highly promising to produce sensors on flexible substrates in large numbers at low cost [34]. Summarizing the production steps could be:

- transducer substrate patterning to have “sticky” and “non-sticky” regions,
- nanowires contact printing,
- deposition of metal contacts and necessary circuitry.

Contact printing is a very simple process, it can be done with different kind and shapes of substrates (planar or cylindrical), it mainly consists in a directional sliding of the substrate, used for the preparation of the structures to transfer, with a receiver substrate. Nanowires alignment and eventual detachment from the growth substrate is due to the shear force. The key parameters of the overall process are nanowires density, if it's too low there is no alignment, the exerted pressure, the contact area between growth and receiver substrate. Last but not least also the nanowires characteristics influence the alignment results, poorly vertically aligned nanowires result in poorly alignment on the receiver substrate. At the end of the process to obtain a full integration in a real functional device, a contacts patterned printing process with standard lithography is required for the needed circuitry.

**Figure 4.**
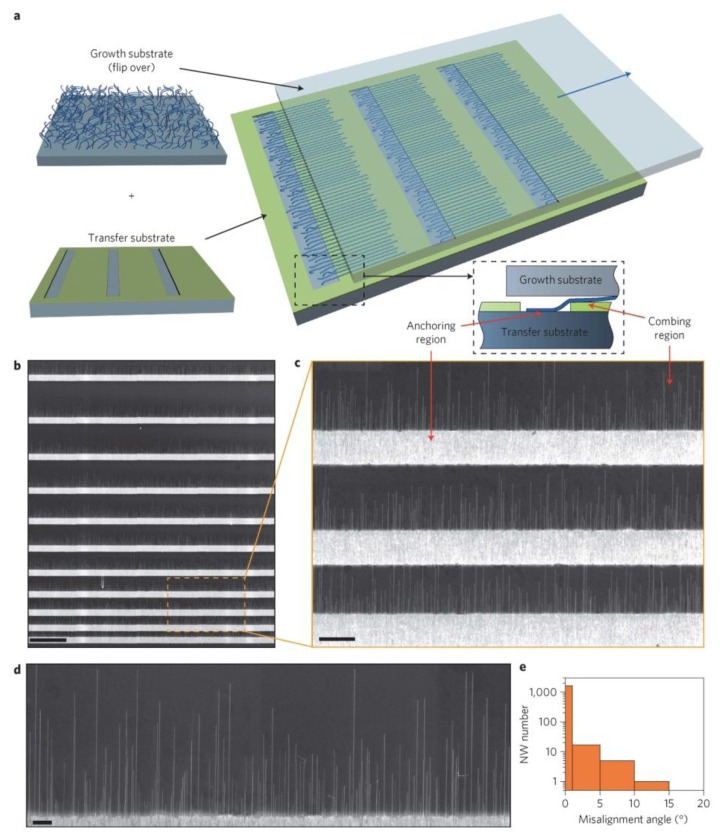
Nanowire device arrays. (**a**) Dark-field image of silicon nanowire arrays. The anchoring windows were defined by photolithography, and the resist layer (S1805) thickness was 70 nm. Scale bar, 100 mm; (**b**) SEM image of one of the combed nanowire arrays on the resist layer. Scale bar, 2 mm; (**c**) Dark-field image of trimmed nanowire arrays (resist layer removed). Scale bar, 40 mm; (**d**) Optical image of nanowire device arrays connecting to electrode arrays. Scale bar, 200 mm; (**e**) Representative SEM image of one of the device arrays. Scale bar, 2 mm; (**f**) I_ds_-V_g_ characteristics (V_ds_ = 0.1 V) from 20 top-gated Ge/Si nanowire devices assembled by nanocombing. The channel length of the devices is 3.8 mm, with Al_2_O_3_ (7 nm) serving as the dielectric layer for the top gate (Cr/Au 1/4 5/50 nm). The electrical characterizations were performed in an ambient environment. Reprinted from [35] by permission from Macmillan Publishers Ltd. Copyright (2013).

Lately a refinement of this process, named nanocombing, has been proposed by Lieber [35] obtaining an alignment of 98.5% within 1° of the combing direction. Figure 4 reports the results obtained with nanocombing for silicon nanowires. This technique has the advantage of providing an array of suspended nanowires and this avoids the detrimental effects of a post process trench etching. Furthermore multi step processes may be performed allowing the achievement of orthogonal nanowires crossbar arrays, that may be interesting for gas sensors development.

## 3. Sensors on Flexible Substrates

Several examples of sensors on flexible substrates have been reported in literature. Most of them use post-growth procedures for the nanowire integration into the sensing device and some are made using a single nanowire.

In 2007 the integration of silicon nanowires by the so-called superlattice nanowire pattern transfer (SNAP) [36] approach into biological and chemical sensors on plastic was reported. The configuration with multiple nanowires is convenient for different reasons. First of all it is easily scalable for industrialization, while the production of single nanowires by nanomanipulation is still not ready for large-scale production. It is very sensitive thanks to the presence of nanowire to nanowire junctions, furthermore there is a smaller effect of the contact resistance influence on the device properties. Inorganic nanowire surfaces, especially silicon and metal oxides, have a well-known termination and chemistry. Moreover they can be easily functionalized to increase their performances. The response to NO_2_ gas diluted in N_2_ was presented with a detection limit of at least 20 ppb. The response was significantly larger than the <1% drift in current for the 10 min before gas delivery. Indeed the measurements were not performed in real conditions (in the presence of humid air and with a measurement of a short and long term drift), nevertheless the results are promising especially for the integration of inorganic nanowires into sensing devices.

From this report a lot of improvements have been achieved concerning the integration, as presented in the previous section, and also the performances of the final device. In 2010 Ahn *et al.* [37] reported on the preparation of ZnO nanorods by thermolysis-assisted chemical solution and the ethanol sensing properties of the prepared sensors on polyimide substrates with performances comparable to the ones reported in literature. The use of flexible substrates does not worsen the sensing capabilities of metal oxide nanowires. The response (defined as the ratio between the resistance in air and with gas) toward 100 ppm of ethanol was 3.11, while response and recovery times were 3–5 min at an operating temperature of 300 °C.

Actually the real problem for the integration in flexible and/or wearable devices, when dealing with metal oxide gas sensors, is the power consumption and their need to operate at relatively high temperatures. Recently several examples of room temperature operation of metal oxide nanowires for the detection of specific gases have been proposed, but almost exclusively on rigid substrates. Fan *et al.* for example showed the ability to achieve a room temperature response towards nitrogen dioxide and ammonia with ZnO field effect transistors prepared on silicon substrates. The response time is relatively slow and to achieve a complete recover of the conductance values in air, after the gas exposure, a high voltage has to be applied to the gate to induce an electrodesorption mechanism.

Electroabsorption effects have been exploited recently in a sensor array in order to increase the selectivity of metal oxides nanowires. Mg-doped indium oxide catalyzed nanowires were integrated into enhanced mode field effect transistors, using the appropriate threshold voltages the single nanowires sensors were able to suppress the non specific sensitivity to carbon monoxide, hydrogen and ethanol at room temperature with response times on the order of seconds. These results may be promising for achievement of room temperature sensing with flexible field effect transistors.

**Figure 5.**
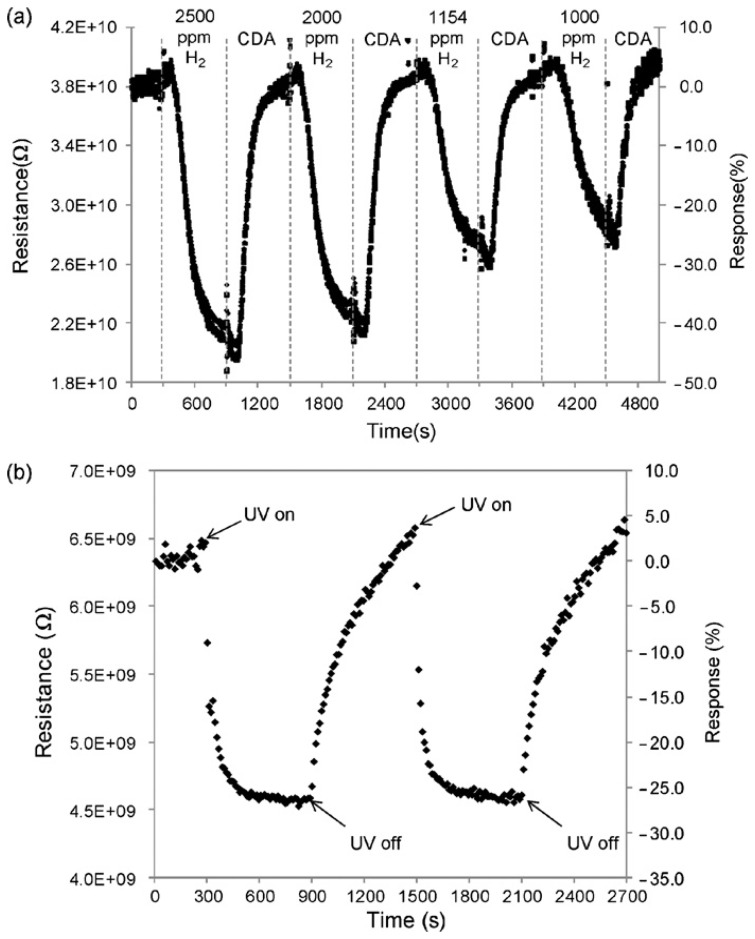
Electrical responses of ZnO NR-on-cloth device to (**a**) various concentrations of H_2_ gas and (**b**) UV irradiation. Clean dry air (CDA) was used to flush the chamber before and after each hydrogen injection. Experiments were performed in room temperature. Reprinted from [27] with permission. Copyright (2010) Elsevier.

In 2010 the integration of ZnO nanorods in multifunctional wearable room temperature sensors was proved [27]. In this case not only were the sensing functional investigations towards hydrogen and UV irradiation performed, but also mechanical tests demonstrating the robustness towards stress and washing cycles. The response towards 500 ppm of hydrogen was about 1.4 at room temperature with response and recovery times of few minutes. The prototype fabric sensors showed promising performances concerning both hydrogen and UV irradiation sensing (Figure 5). Furthermore ZnO nanogenerators (proposed by Wang *et al.* [38]) may be incorporated into the prototypes to achieve a self-powered wearable sensor.

Indeed, the more interesting options are the ones that exploit the peculiarities of nanowires that may lead to essential progress towards autonomous and distributed gas sensors networks. For example, the interesting self-heating strategy for chemical gas sensor development was proposed on rigid substrates [39], but may be easily integrated into flexible devices using nanocombing techniques. The proposed gas sensor device was based on the self-heating process of a single nanowire due to the dissipated power (Joule effect) induced by the bias current applied in conductometric measurements. Thanks to its small mass, the nanowire was heated up to the working temperatures needed for gas sensing applications with few tens of microW. Moreover using a single nanowire should reduce the thermal inertia of the sensors, minimize gas diffusion processes, resulting in a fast dynamic responses only limited by the surface reaction kinetics. Combining low power electronics with continuous and pulsed self-heating of nanowires, power consumptions in the microwatt range or even lower may be achieved [40].

Another strategy to reduce the operating temperature, that may be integrated into flexible substrates, is optical excitation, that has also the advantage of improving the adsorption desorption processes. Exciting the metal oxide semiconductor with photons above the band gap produces free carriers in the space charge region and in an n-type semiconductor excess holes are attracted towards the surface and electrons are swept away from the surface and therefore surface band bending is decreased. This plays a significant role in the detection of adsorbed gas species influencing chemisorption processes, as reported several years ago for polycrystalline metal oxide chemical sensors [41–44]. Law *et al.* [45] demonstrated the possibility of using individual nanowires for UV optical excited gas sensing applications. Afterwards in [46] an interesting comparison between the response of optical excited single nanowire devices and the one obtained operating at higher temperatures was reported, showing the possibility to use light instead of temperature activation. Furthermore optical excitation can be performed by illumination of nanowires with commercial UV or blue light LEDs leading to cheaper sensing platforms.

These results may have implications in the use of sensors for applications that range from real-time pollution regulation to highly portable biological- and chemical-threat detectors. Furthermore, the low power/heat dissipation and high sensitivity of these devices coupled with inherent biocompatibility of the plastic substrates may have exiting applications *in vivo* biomolecular monitoring.

A key issue that has not been well addressed in the field of nanowire flexible gas sensors is their stability, Courbat *et al.* [47,48] have reported on the continuous operation of metal oxide polycrystalline gas sensors on polyimide hotplates for several months, so comparable results may be expected for nanowires based devices. Other issues that have still not been sufficiently explored include production yield and reliability, especially under mechanical deformation conditions.

## 4. Conclusions

The support for nanotechnology is still firm and intense in Europe and the US, but the focus has been moved to its possible practical applications. The first enthusiasm has now given way to the doubts and challenges due to economic crisis, energy, climate change, health care and national security issues. The key point to address the real applications of nanowires in sensing devices is the ability to consider the full problem and not only a specific, as all the aspects of this multifaceted problem must be addressed to optimize the final device and achieve large scale production of fully functional devices. Nanotechnology still promises to contribute and improve all the above, but now there is the need to make products that realize this promise. The real success of nanotechnology is strictly related to its ease of manufacturing nanomaterials and their effective integration into multifunctional devices for large-scale production and commercialization.

## Figures and Tables

**Table 1.** Top ten most cited articles in the topic nanowire* sensor* or nanorod* sensor* or nanobelt* sensor* (IsiWebofKnowledge, 10 June 2013).

**Ranking**	**Title**	**Total Citations**	**Average Citations Per Year**	**Reference**
**1**	Nanowire nanosensors for highly sensitive and selective detection of biological and chemical species	2,970	228	[13]
**2**	Growth of nanowire superlattice structures for nanoscale photonics and electronics	1,539	128	[14]
**3**	Coaxial silicon nanowires as solar cells and nanoelectronic power sources	1,089	157	[15]
**4**	Zinc oxide nanostructures: growth, properties and applications	998	100	[16]
**5**	Multiplexed electrical detection of cancer markers with nanowire sensor arrays	950	106	[17]
**6**	Carbon nanotubes: Synthesis, integration, and properties	864	72	[18]
**7**	Fabrication and ethanol sensing characteristics of ZnO nanowire gas sensors	861	86	[19]
**8**	Large-scale hexagonal-patterned growth of aligned ZnO nanorods for nano-optoelectronics and nanosensor arrays	829	83	[20]
**9**	Stable and highly sensitive gas sensors based on semiconducting oxide nanobelts	810	67	[21]
**10**	Hydrogen sensors and switches from electrodeposited palladium mesowire arrays	747	57	[22]
